# 

**Published:** 1960-10

**Authors:** 


### THE MEDICAL JOURNAL OF THE SOUTH-WEST

*Incorporating*
*The Bristol Medico-Chirurgical Journal*

NATIONAL LIBRARY OF MEDICINE
NOV 23 1960

*October 1960*

VOL. 75 (iv). No. 278

